# Association between timing and type of postnatal care provided with neonatal mortality: A large scale study from India

**DOI:** 10.1371/journal.pone.0272734

**Published:** 2022-09-16

**Authors:** Lucky Singh, Ritam Dubey, Prashant Kumar Singh, Saritha Nair, M. Vishnu Vardhana Rao, Shalini Singh

**Affiliations:** 1 ICMR National Institute of Medical Statistics, Ansari Nagar, New Delhi, India; 2 Division of Preventive Oncology and Population Health, ICMR National Institute of Cancer Prevention and Research, Noida, Uttar Pradesh, India; Regional Medical Research Centre Bhubaneswar, INDIA

## Abstract

**Objectives:**

This study examines the association between quality Postnatal Care (PNC) considering timing and providers’ type on neonatal mortality. The aim extends to account for regional disparities in service delivery and mortality including high and non-high focus states.

**Methods:**

Ever-married women aged 15–49 years (1,87,702) who had delivered at least one child in five years preceding the survey date surveyed in National Family Health Survey (2015–16) were included in the study. Neonatal deaths between day two and seven and neonatal deaths between day two and twenty-eight were considered dependent variables. Descriptive statistics and multivariate regression analysis were conducted.

**Results:**

Chances of early neonatal mortality were 29% (OR = 0.71; 95%CI: 0.59–0.84) among newborns receiving PNC within a day compared to ones devoid of it while 40% (OR: 0.60; 95%CI: 0.51–0.71) likelihood for the same was noted if PNC was delivered within a week. Likelihood of neonatal mortality decreased by 24% (OR: 0.76; 95%CI: 0.65–0.88) when skilled PNC was delivered within 24 hours. Receiving quality PNC by skilled providers within a day in a non-high focus state decreased the chances of neonatal mortality by 26% (OR: 0.74; 95%CI: 0.59–0.92) compared to ones who did not receive any PNC.

**Conclusions:**

Neonatal deaths were significantly associated with socioeconomic and contextual characteristics including age, education, household wealth, social group and region. Timing of PNC delivered and by a skilled healthcare provider was found significant in reducing neonatal mortality.

## Introduction

Postnatal period is crucial for the lives of both the newborn and the mother where about 40 per cent of the women face post-delivery complications [1]: about 99 per cent of 2.6 million neonatal deaths occur in low-and-middle income countries (LMICs) alone [2]. The neonatal period has been noted with the slowest pace of improvement especially among the LMICs [3] where 36 per cent of 2.5 million newborn deaths in 2017 occurred on the same day of their birth [4] and about 75 per cent of neonatal deaths occurred within one week of birth [5].

Recent data from India indicates 30 neonatal deaths per 1000 live births [6] where about 80 per cent are early neonatal deaths [7]. Among the many causes of neonatal deaths preterm births, neonatal infection, intra-partum related complications (including, birth asphyxia) and congenital malformations [8] occupy a major share. Fewer prenatal visits [9], low birth weight [10] delivery at home not followed by postnatal care [11] and absence of skilled birth attendance [8] have also been found to be associated with early neonatal and neonatal deaths.

According to WHO’s latest reports, neonatal deaths are associated more with maternal health compared to other under-five deaths; these are preventable with timely and sufficient interventions along the continuum of care, including during the postnatal period [7]. The Government of India (GoI) has mandated Postnatal Care (PNC) services (including checkup of the newborn) to be provided within a day of delivery and in subsequent four home visits till the 42^nd^ day by trained health providers [12], in line with guidelines suggested by the WHO [7]. Although relevant PNC policies for both mother and newborn are in place, the focus on its content, quality and outreach has long been overlooked when compared to antenatal and delivery care [4]. The SDG set by the United Nations [13] to reduce neonatal mortality to 12 per 1000 live births is a far cry from present statistics.

This study explores the aspects of early obstetric services available to the newborn through well-known conceptual frameworks such as Mosley and Chen’s [14] and Framework of Health Services Utilization by Andersen and Newman [15]. The former framework is utilized to examine socio-economic factors associated with child survival in India. The latter framework was considered as it viewed health status (here, neonatal mortality) as an outcome of intake or inability to utilize present healthcare services (postnatal services for the newborn in the present study) affected by varied factors—environmental, population-related and health-seeking behaviour. GoI has identified Bihar, Madhya Pradesh, Orissa, Chhattisgarh, Jharkhand, Rajasthan, Uttarakhand, Uttar Pradesh and Assam, as ‘high focus group states’ (HFGS) to provide more focused attention to improving the overall health performance of these states [16] and remaining were categorized as non-high focus group states.

It is imperative to learn the nuances through which the before-mentioned socio-economic factors operate in these specific regions compared to Non- High Focus Group states (NHFGS) of India to inform future health policy initiatives. The objective of the present study is to examine the association of timely postnatal services varying by provider type with early neonatal and neonatal deaths, especially among High and Non-High Focus states of India.

## Methods

### Data

Cross-sectional data analysis was conducted on the data obtained from National Family Health Survey (NFHS-4) held in India during 2015–16 [6]. A nationally representative survey, NFHS-4 was carried out in 29 states and 7 union territories of India. A total of 1,87,702 women aged 15–49 years who were noted to be married anytime in their lives and had delivered at least one child during the five years preceding the survey were included in the study.

### Dependent variables

Study considered two dependent variables in the analysis—neonatal death between day two and day seven and neonatal death between day two and day twenty-eight. The first outcome variable is referred to as early neonatal death and the latter one as neonatal death. The analysis for neonatal deaths did not include all the neonates born within one month as the survey does not completely capture the timing of death that is if the newborn died before or after being eligible for the postnatal care. Hence, by the literature attempting a similar analysis [17] neonates surviving the first 24 hours were included to eliminate the possibility of censoring in the data analysis.

### Independent variables

The study included varied individual, household and programme level factors in accordance with their theoretical and empirical significance highlighted in the literature. In the survey, questions posed regarding PNC for newborns to the women were,

“*How many hours, days or weeks after the birth did the first check up take place?*”

“*Who checked the baby’s health at that time?*”

Responses from these questions combined with the type of provider (skilled/unskilled) for delivery have been used in the study to construct two separate variables for PNC from day one to day seven.

1. Skilled PNC (checked by physicians, nurses or midwives)
2. Unskilled PNC (checked by Traditional Birth Attendants or community health workers)
3. Did not access or received any PNC on Day One or by Day Seven.

Study included other maternal healthcare factors that are shown to be significantly associated with child health outcomes both as conceptual frameworks described above and in recent literature [18, 19], such as frequency of antenatal visits during pregnancy (categorised as< 4 and > = 4 visits), reception of tetanus toxoid (TT) injection during pregnancy (yes/ no), specifics of delivery (delivery by unskilled healthcare staff at home, delivery by unskilled healthcare staff in a health facility, delivery by skilled healthcare staff at home or delivery by skilled healthcare staff in a facility), low birth weight (that is weight < 2.5 kg) (yes/no) and caesarean delivery (yes/no).

The study considered a spectrum of socioeconomic and demographic factors such as the age of the mother at the time of birth, women’s parity, women’s education, low birth weight and gender composition of living children. Self-reported identification of religion was divided into Hindu, Muslim and Other religions (Sikh, Christians and Jains) and castes were grouped into Others, Scheduled Tribes (STs), Scheduled Castes (SCs) and Other Backward Classes (OBCs). Household wealth, urban-rural residence and region were also considered in the study.

### Statistical analysis

At first, the sample distribution of all the births that occurred five years before the date of the survey was presented by selected and relevant contextual and background characteristics. Both unadjusted and adjusted binary logistic regression modeling approach was used to evaluate the relationship between the early neonatal deaths and their possible predictors. Considering the substantial difference in neonatal mortality across the states of India, binary regression analysis was carried out between variables of types of PNC provided (none, unskilled and skilled) and states categorized under High and Non-High Focus states. Data analyses were performed using the Stata 12 statistical software [20].

### Ethics statement

Patients and/or the participants were not involved in the development of research questions, design, or conduct/ reporting/ dissemination plans of this research as this study involves secondary research of the data collected in the NFHS-4 survey. The information collected in the NFHS-4 was used primarily for research where the personal identifiers were not disclosed and informed consent was obtained before the conduction of the survey.

## Results

### Description of the sample distribution

Sample distribution of women considered in the present study has been summarized in Table 1. Nearly, 8% of the women delivered during adolescence (less than 19 years of age). Majority of women (42%) in the study belonged to the age group 20–24 years. Over one in every four women were illiterate (28%) and about one in five had completed 12 years of schooling and more. Out of all births that were included in the study, 17% recorded low birth weight and nearly one in every five was caesarean delivery. Three in every five (75%) and about 69% of the women did not visit for PNC on day one and day seven, respectively.

**Table 1:** Sample distribution of women with birth during five years preceding the survey, National Family Health Survey (NFHS) 2015–16, India.

Background Characteristics	*n*	%
**Age at birth (years)**		
< = 19	13608	8.1
20–24	74084	41.8
25–29	61535	32.4
30–34	26344	12.5
35–39	9221	3.9
40–49	2910	1.2
**Women’s education**		
No schooling	54352	27.7
Less than 5years	11491	5.8
5 to 7 years	29444	15.9
8 to 9 years	34018	16.8
10 to 11 years	21828	12.6
12 or more years	36569	21.2
**Parity**		
1	60689	33.6
2 to 3	94137	51.2
4 to5	24412	11.4
6 and above	8464	3.8
**Gender composition of living children**		
No sons	50718	27.7
At least one son	136984	72.3
**Wealth quintile**		
Poorest	45769	23.2
Poorer	42919	21.1
Middle	37769	19.9
Richer	32804	19.1
Richest	28441	16.7
**Caste**		
Others	34236	21.4
Scheduled Castes	34476	22.1
Scheduled Tribes	37245	10.8
Other Backward Classes	72799	45.7
**Religion**		
Hindu	135985	78.9
Muslim	28779	16.1
Others	22938	5.0
**Low birth weight**	24547	17.4
**C-section delivery**	29349	19.3
**Antenatal**		
Four or more ANC visits	88172	51.4
Tetanus toxoid before or during pregnancy	174507	93.7
**Delivery**		
Unskilled at home	33855	14.7
Unskilled in a facility	3194	1.9
Skilled at home	8074	4.2
Skilled in a facility	142579	79.2
**Postnatal**		
***Visit on Day 1***		
None	143149	74.7
Unskilled	4607	2.2
Skilled	39946	23.1
***Visit by Day 7***		
None	131427	68.5
Unskilled	9728	4.5
Skilled	46547	27.0
**Place of residence**		
Rural	140490	70.1
Urban	47212	29.9
**Region**		
Non high focused states	77391	47.5
High focused states	110311	52.5
**Total**	**187702**	**100**

Note: All *’n’* are unweighted

### Unadjusted association

Results presented in Table 2 describes that the odds of death in the first week were lower among children who had skilled PNC on day one (UOR: 0.59; 95%CI: 0.50–0.70) as compared with those who never had any PNC. Similarly, the likelihood of early neonatal mortality was lower among children who utilized either unskilled (UOR: 0.39; 95%CI:0.26–0.58) or skilled delivery of PNC (UOR:0.50;95%CI:0.42–0.59) within seven days after birth than among children who never received PNC by the seventh day. The findings regarding neonatal mortality suggests lower odds of death among children who received skilled PNC on day one after birth as compared with those children who never received PNC.

**Table 2:** Parameter estimates (Unadjusted Odds Ratio (UOR)) for binary weighted logistic regression models of neonate deaths between Days 2 and 7 and deaths between Days 2 and 28 by PNC and other selected background variables, India 2015–16.

Explanatory Variables	Deaths in the First Week (2 to 7 days)				Deaths in the First Month (2 to 28 days)			
	UOR	p-value	95% CI		UOR	p-value	95% CI	
**Postnatal Care visit on Day 1**								
None (ref.)	1.00				1.00			
Unskilled	0.66	0.074	0.41	1.04	0.67	0.045	0.46	0.99
Skilled	0.59	0.000	0.50	0.70	0.62	0.000	0.54	0.72
**Postnatal care visit by Day 7**								
None (ref.)	1.00				1.00			
Unskilled	0.39	0.000	0.26	0.58	0.49	0.000	0.38	0.63
Skilled	0.50	0.000	0.42	0.59	0.60	0.000	0.54	0.67
**Antenatal visits**								
Less than 4 (ref.)	1.00				1.00			
Four or more	0.70	0.000	0.63	0.77	0.69	0.000	0.63	0.75
**Tetanus toxoid injection before or during pregnancy**								
No (ref.)	1.00				1.00			
Yes	0.66	0.000	0.53	0.81	0.65	0.000	0.54	0.79
**Delivery**								
Unskilled at home (ref.)	1.00				1.00			
Unskilled in a facility	0.53	0.006	0.33	0.83	0.53	0.002	0.35	0.79
Skilled at home	0.56	0.000	0.41	0.76	0.65	0.003	0.49	0.87
Skilled in a facility	0.56	0.000	0.49	0.65	0.54	0.000	0.47	0.61
**Low birth weight**								
No (ref.)	1.00				1.00			
Yes	3.03	0.000	2.57	3.57	3.33	0.000	2.98	3.72
**C-section delivery**								
No (ref.)	1.00				1.00			
Yes	0.81	0.016	0.68	0.96	0.79	0.002	0.68	0.92
**Age at birth**								
< = 19 (ref.)	1.00				1.00			
20–24	0.84	0.122	0.67	1.05	0.76	0.004	0.62	0.92
25–29	0.78	0.039	0.62	0.99	0.80	0.028	0.65	0.98
30–34	1.05	0.714	0.81	1.35	1.04	0.695	0.84	1.29
35–39	1.37	0.038	1.02	1.85	1.30	0.048	1.00	1.69
40–49	1.96	0.000	1.35	2.85	2.09	0.000	1.53	2.87
**Women’s education**								
No schooling (ref.)	1.00				1.00			
Less than 5years	0.94	0.615	0.74	1.20	0.87	0.196	0.70	1.08
5 to 7 years	0.79	0.007	0.67	0.94	0.76	0.001	0.65	0.89
8 to 9 years	0.73	0.001	0.60	0.87	0.72	0.000	0.62	0.85
10 to 11 years	0.48	0.000	0.38	0.60	0.43	0.000	0.35	0.53
12 or more years	0.40	0.000	0.32	0.49	0.39	0.000	0.32	0.46
**Parity**								
1 (ref.)	1.00				1.00			
2 to 3	0.79	0.002	0.69	0.92	0.84	0.009	0.74	0.96
4 to5	1.36	0.001	1.14	1.63	1.49	0.000	1.27	1.74
6 and above	2.14	0.000	1.72	2.66	2.38	0.000	1.97	2.86
**Gender composition of living children**								
No sons (ref.)	1.00				1.00			
At least one son	0.27	0.000	0.24	0.31	0.28	0.000	0.25	0.31
**Wealth quintile**								
Poorest (ref.)	1.00				1.00			
Poorer	0.80	0.003	0.68	0.93	0.78	0.000	0.68	0.89
Middle	0.67	0.000	0.56	0.79	0.65	0.000	0.56	0.77
Richer	0.42	0.000	0.35	0.52	0.41	0.000	0.34	0.49
Richest	0.34	0.000	0.27	0.43	0.33	0.000	0.27	0.41
**Caste**								
Others (ref.)	1.00				1.00			
Scheduled Castes	1.45	0.001	1.17	1.79	1.45	0.000	1.20	1.75
Scheduled Tribes	1.37	0.010	1.08	1.73	1.34	0.007	1.08	1.65
Other Backward Classes	1.23	0.040	1.01	1.49	1.22	0.023	1.03	1.46
**Religion**								
Hindu (ref.)	1.00				1.00			
Muslim	0.90	0.215	0.76	1.06	0.87	0.076	0.75	1.01
Others	0.68	0.012	0.50	0.92	0.65	0.001	0.50	0.84
**Place of residence**								
Rural (ref.)	1.00				1.00			
Urban	0.70	0.000	0.62	0.78	0.58	0.000	0.49	0.67
**Region**								
Non high focused states (ref.)	1.00				1.00			
High focused states	2.08	0.000	1.79	2.42	2.23	0.000	1.95	2.55

### Adjusted association

#### PNC and early neonatal mortality

Model 1 shows 27 per cent (OR:0.73;95%CI:0.64–0.83) lower likelihood of early neonatal mortality among children who had utilized skilled PNC on day one as compared to those children who did not use any PNC (Table 3). After adjusting for all the selected background variables in Model 2, the findings confirm lower odds of early neonatal death among children who used skilled PNC (OR:0.71;95%CI:0.59–0.84) on day one than neonates who did not access any PNC. A similar pattern was observed for PNC utilization by day seven and its effect on early neonatal mortality. After adjusting for all the independent variables in Model 4, PNC use by day seven had a significant effect on reduction of early neonatal death compared to the absence of the same where unskilled PNC (OR:0.37; 95%CI:0.25–0.55) was found with higher likelihood early neonatal survival compared to skilled PNC (OR:0.60;95%CI:0.51–0.71).

**Table 3:** Parameter estimates (Odds Ratio) for binary weighted logistic regression models of neonate deaths between Days 2 and 7 by PNC types and other selected background variables (N = 187702) India, 2015–16.

Explanatory Variables	Model 1				Model 2				Model 3				Model 4			
	OR	p-value	95% CI		OR	p-value	95% CI		OR	p-value	95% CI		OR	p-value	95% CI	
**Postnatal care visit on day 1**																
None (ref.)	1.00				1.00											
Unskilled	0.71	0.056	0.50	1.01	0.58	0.021	0.36	0.92								
Skilled	0.73	0.000	0.64	0.83	0.71	0.000	0.59	0.84								
**Postnatal care visit by day 7**																
None (ref.)									1.00				1.00			
Unskilled									0.46	0.000	0.34	0.62	0.37	0.000	0.25	0.55
Skilled									0.62	0.000	0.55	0.71	0.60	0.000	0.51	0.71

Model 1: Postnatal care visit on day 1 + antenatal care visit + received TT injection during pregnancy + place of delivery + C-section delivery + low birth weight.

Model 2: Postnatal care visit on day 1 + antenatal care visit + received TT injection during pregnancy + place of delivery + C-section delivery + low birth weight + mother’s age at birth + mother’s schooling + parity + gender composition of living children + household wealth + social group (caste) + religion + place of residence + state.

Model 3: Postnatal care visit on day 7 + similar as Model 1.

Model 4: Postnatal care visit on day 7 + similar as Model 2.

### Adjusted association

#### PNC and neonatal mortality

Results revealed lower odds of neonatal mortality among children who received either unskilled (OR:0.62;95% CI:0.42–0.92) or skilled (OR:0.76;95%CI:0.65–0.88) PNC by day one than among those who did not receive PNC in Model 1 (Table 4). Even after adjusting for other independent variables in Model 2, the pattern of association between PNC care by unskilled and skilled providers on day one and neonatal mortality was noted to be similar. The odds of neonatal mortality was lower among children who received unskilled (OR:0.42;95%CI:0.31–0.57) and skilled (OR:0.66;95%CI:0.57–0.76) PNC in comparison to those who did not receive PNC by day seven, after controlling for all the selected independent variables (Model 4).

**Table 4:** Parameter estimates (Odds Ratio) for binary weighted logistic regression models of neonate deaths between Days 2 and 28 by PNC type and other selected background variables, India 2015–16.

Explanatory Variables	Model 1				Model 2				Model 3				Model 4			
	OR	p-value	95% CI		OR	p-value	95% CI		OR	p-value	95% CI		OR	p-value	95% CI	
**Postnatal care visit on Day 1**																
None (ref.)	1.00				1.00											
Unskilled	0.62	0.016	0.42	0.92	0.57	0.005	0.38	0.84								
Skilled	0.76	0.000	0.65	0.88	0.76	0.000	0.65	0.88								
**Postnatal care visit by Day 7**																
None (ref.)									1.00				1.00			
Unskilled									0.46	0.000	0.34	0.63	0.42	0.000	0.31	0.57
Skilled									0.65	0.000	0.57	0.75	0.66	0.000	0.57	0.76

Model 1: Postnatal care visit on day 1 + antenatal care visit + received TT injection during pregnancy + place of delivery + C-section delivery + low birth weight.

Model 2: Postnatal care visit on day 1 + antenatal care visit + received TT injection during pregnancy + place of delivery + C-section delivery + low birth weight + mother’s age at birth + mother’s schooling + parity + gender composition of living children + household wealth + social group (caste) + religion + place of residence + state.

Model 3: Postnatal care visit on day 7 + similar as Model 1.

Model 4: Postnatal care visit on day 7 + similar as Model 2

### Interaction effect

#### PNC × state

Table 5 presents the results of the interaction effect between type of PNC utilization and states grouped into two categories, HFGS and NHFGS. As compared to children who did not utilize PNC on day one in NHFGS, the odds of early neonatal mortality were 41 per cent (OR: 1.41; 95%CI:1.15–1.72) high among children of HFGS who were devoid of PNC. The likelihood of early neonatal death was significantly lower among children belonging to NHFGS who utilized unskilled PNC on day one as compared to children of NHFGS and those who did not use PNC (OR:0.14;95%CI:0.04–0.46). Further, 45 per cent (95%CI:1.26–1.67) higher likelihood of early neonatal death was noted among children who did not utilize PNC by Day seven in HFGS as compared to NHFGS children. As compared to children who were from NHFGS and never used PNC by day seven, the likelihood of early neonatal death was significantly lower among children who used unskilled and skilled PNC and belonged to NHFGS and HFGS.

**Table 5:** Binary weighted logistic regression models showing interaction effect between PNC and regions on child deaths, India 2015–16.

Explanatory variables	2–7 days mortality				2–7 days mortality				2–28 days mortality				2–28 days mortality			
	OR	p-value	95% CI		OR	p-value	95% CI		OR	p-value	95% CI		OR	p-value	95% CI	
PNC on day 1 * Regions																
None*non-HFGS (ref.)	1.00								1.00							
None*HFGS	1.41	0.001	1.15	1.72					1.50	0.000	1.33	1.70				
Unskilled*non-HFGS	0.14	0.001	0.04	0.46					0.77	0.477	0.38	1.57				
Unskilled*HFGS	0.94	0.812	0.56	1.57					0.98	0.890	0.69	1.39				
Skilled*non-HFGS	0.58	0.003	0.40	0.83					0.74	0.007	0.59	0.92				
Skilled*HFGS	1.11	0.380	0.88	1.41					1.18	0.045	1.00	1.38				
PNC by day 7 * Regions																
None*non-HFGS (ref.)					1.00								1.00			
None*HFGS					1.45	0.000	1.26	1.67					1.47	0.000	1.22	1.76
Unskilled*non-HFGS					0.48	0.018	0.26	0.88					0.29	0.003	0.13	0.66
Unskilled*HFGS					0.60	0.005	0.42	0.85					0.67	0.035	0.46	0.97
Skilled*non-HFGS					0.54	0.000	0.42	0.69					0.51	0.000	0.37	0.68
Skilled*HFGS					0.93	0.438	0.78	1.12					1.11	0.338	0.90	1.36
**Antenatal visits**																
Less than 4 (ref.)	1.00				1.00				1.00				1.00			
Four or more	1.03	0.726	0.88	1.20	1.10	0.106	0.98	1.23	1.05	0.339	0.95	1.16	1.02	0.728	0.89	1.17
**Tetanus toxoid before or during pregnancy**																
No (ref.)	1.00				1.00				1.00				1.00			
Yes	0.87	0.230	0.69	1.09	0.97	0.742	0.82	1.15	0.93	0.340	0.80	1.08	0.89	0.249	0.73	1.08
**Delivery**																
Unskilled at home (ref.)	1.00				1.00				1.00				1.00			
Unskilled in a facility	1.28	0.304	0.80	2.06	1.50	0.037	1.02	2.18	1.37	0.073	0.97	1.93	1.18	0.447	0.77	1.81
Skilled at home	0.88	0.416	0.63	1.21	0.99	0.931	0.76	1.28	1.02	0.833	0.82	1.28	1.01	0.945	0.75	1.35
Skilled in a facility	1.74	0.000	1.41	2.15	1.79	0.000	1.54	2.07	1.65	0.000	1.45	1.89	1.55	0.000	1.27	1.90
**Low birth weight**																
No (ref.)	1.00				1.00				1.00				1.00			
Yes	2.74	0.000	2.31	3.24	2.76	0.000	2.43	3.14	2.95	0.000	2.64	3.30	2.96	0.000	2.55	3.43
**C-section delivery**																
No (ref.)	1.00				1.00				1.00				1.00			
Yes	1.33	0.006	1.08	1.62	1.35	0.000	1.17	1.56	1.37	0.000	1.21	1.56	1.33	0.002	1.11	1.59
**Age at birth (years)**																
< = 19 (ref.)	1.00				1.00				1.00				1.00			
20–24	1.01	0.948	0.79	1.28	1.00	0.969	0.83	1.20	0.89	0.174	0.76	1.05	0.89	0.245	0.72	1.09
25–29	1.00	0.977	0.77	1.29	1.02	0.841	0.83	1.25	0.96	0.658	0.81	1.15	0.98	0.860	0.78	1.22
30–34	1.13	0.406	0.84	1.52	1.23	0.074	0.98	1.55	1.14	0.197	0.93	1.40	1.09	0.498	0.85	1.40
35–39	1.24	0.234	0.87	1.76	1.44	0.009	1.09	1.90	1.23	0.104	0.96	1.57	1.12	0.483	0.82	1.53
40–49	1.46	0.095	0.94	2.26	1.76	0.002	1.22	2.53	1.63	0.002	1.19	2.24	1.43	0.067	0.98	2.09
**Women’s education**																
No schooling (ref.)	1.00				1.00				1.00				1.00			
Less than 5years	1.21	0.129	0.95	1.56	1.27	0.015	1.05	1.54	1.20	0.036	1.01	1.43	1.14	0.240	0.91	1.43
5 to 7 years	1.15	0.157	0.95	1.38	1.17	0.040	1.01	1.35	1.14	0.054	1.00	1.30	1.13	0.172	0.95	1.34
8 to 9 years	1.04	0.684	0.85	1.28	1.08	0.352	0.92	1.25	1.06	0.430	0.92	1.21	1.07	0.465	0.89	1.28
10 to 11 years	0.81	0.119	0.62	1.06	0.97	0.758	0.80	1.18	0.93	0.438	0.78	1.11	0.77	0.031	0.61	0.98
12 or more years	0.73	0.023	0.56	0.96	0.82	0.042	0.67	0.99	0.79	0.009	0.67	0.94	0.74	0.011	0.58	0.93
**Parity**																
1 (ref.)	1.00				1.00				1.00				1.00			
2 to 3	1.10	0.251	0.94	1.29	1.08	0.197	0.96	1.23	1.14	0.022	1.02	1.27	1.17	0.026	1.02	1.35
4 to5	1.66	0.000	1.32	2.10	1.58	0.000	1.32	1.90	1.72	0.000	1.47	2.03	1.78	0.000	1.44	2.19
6 and above	2.25	0.000	1.64	3.07	2.14	0.000	1.67	2.74	2.44	0.000	1.96	3.03	2.48	0.000	1.89	3.26
**Gender composition of living children**																
No sons (ref.)	1.00				1.00				1.00				1.00			
At least one son	0.20	0.000	0.18	0.23	0.20	0.000	0.18	0.22	0.19	0.000	0.18	0.21	0.19	0.000	0.17	0.22
**Wealth quintile**																
Poorest (ref.)	1.00				1.00				1.00				1.00			
Poorer	0.98	0.780	0.83	1.15	1.05	0.485	0.92	1.19	1.01	0.859	0.90	1.13	0.98	0.793	0.85	1.13
Middle	1.01	0.893	0.82	1.25	0.96	0.560	0.82	1.11	0.94	0.412	0.82	1.08	1.03	0.761	0.86	1.23
Richer	0.76	0.054	0.58	1.00	0.75	0.003	0.62	0.91	0.73	0.000	0.61	0.86	0.74	0.018	0.58	0.95
Richest	0.76	0.143	0.52	1.10	0.73	0.008	0.57	0.92	0.74	0.005	0.60	0.91	0.74	0.067	0.54	1.02
**Caste**																
Others (ref.)	1.00				1.00				1.00				1.00			
Scheduled Castes	1.05	0.663	0.84	1.32	1.30	0.002	1.10	1.54	1.23	0.005	1.06	1.42	1.05	0.672	0.85	1.29
Scheduled Tribes	0.93	0.594	0.72	1.21	1.04	0.690	0.86	1.25	0.98	0.768	0.83	1.15	0.91	0.415	0.72	1.15
Other Backward Class	0.95	0.630	0.77	1.17	1.08	0.323	0.93	1.25	1.03	0.669	0.90	1.17	0.94	0.505	0.78	1.13
**Religion**																
Hindu (ref.)	1.00				1.00				1.00				1.00			
Muslim	0.90	0.272	0.74	1.09	1.04	0.580	0.90	1.20	1.02	0.719	0.90	1.16	0.85	0.072	0.72	1.01
Others	0.95	0.747	0.69	1.31	0.78	0.022	0.63	0.96	0.73	0.001	0.60	0.89	0.93	0.598	0.70	1.23
**Place of residence**																
Rural (ref.)	1.00				1.00				1.00				1.00			
Urban	0.82	0.072	0.66	1.02	0.91	0.193	0.79	1.05	0.94	0.291	0.83	1.06	0.88	0.190	0.73	1.06

Note: HFGS: High Focus Group States

A similar pattern was evident in the case of neonatal mortality. The odds of neonatal death were 50 per cent higher among children in HFGS who did not utilize PNC on Day one as compared to children in NHFGS (OR:1.50;95%CI:1.33–1.70). The likelihood of neonatal mortality was 26 per cent (OR: 0.74; 95%CI:0.59–0.92) lower among children who had skilled PNC on day one after delivery and belonged to NHFGS than among children who never had PNC and lived in the same region. Lower likelihood of neonatal mortality was evident in the case of those children who had unskilled and skilled PNC and belonged to HFGS and NHFGS as compared to children of NHFGS and who did not use PNC by Day 7.

## Discussion

Findings revealed that both skilled and unskilled PNC has a significant association with lower chances of neonatal deaths as compared to no care. Knowledge of healthcare workers (skilled and unskilled) has been noted to be associated with the utilization of services, for instance, administration of Tetanus Toxoid injections during the prenatal period and Kangaroo Mother Care (KMC) of the newborn [21].

Interestingly, unadjusted and adjusted odds ratios estimates highlighted that PNC delivered within the first week through unskilled healthcare workers fared better than PNC provided by skilled personnel with regards to fewer chances of neonatal deaths. In concurrence, skilled delivery at a facility was found to have higher chances of neonatal mortality for both early neonatal and neonatal periods. A recent study concurs that the persistent less than adequate delivery of health services in 137 LMICs (including India) is resulting in more neonatal deaths than non-utilization of services [22]. The findings of the study explains the disproportionate dealing of complicated maternal and newborn cases by skilled personnel as noted by recent studies where the majority are not dealt with by doctors necessarily [22, 23]. The debatable competence of the skilled healthcare workers combined with the lack of infrastructure especially related to essential newborn care at healthcare facilities [24] explains this unexpected trend. Significant implications for the quality of care received by the newborn should be reviewed by future studies under the context of overcrowding and early discharge [25].

Analysis conducted on the PNC provided on day one and the first week among high and non-high focus states showed that chances of neonatal deaths are more likely to occur in high focus states by a margin of more than 50 per cent by both the first week and by the end of the first month. Unskilled PNC provided by day one or in the first week in high focus regions of the country was found to be decreasing the likelihood of neonatal mortality when compared to newborns devoid of PNC in the non-high focus states. Skilled health personnel have been noted to attend to more complicated cases during delivery and post natal period [26] and the finding calls for further explanation of the skewed number of such adverse pregnancy outcomes through qualitative approaches. A recent review of newborn care in the high focus states shed light on the dismal pace at which PNC services were made available to newborns [27]. Two recent studies have noted about half of all deliveries were monitored by unqualified personnel and a range of essential clinical practices were rarely observed [28, 29]. Similarly, Kangaroo Mother Care (KMC) has been found operationally effective in Assam, but Jharkhand and Uttarakhand lack awareness about KMC; Sick Newborn Care Units have not been established in these states [30]. States like Bihar and Chattisgarh have infrastructural deficiency including human resources possibly explaining the findings of the present study. However, in the context of non-high focus states, it was observed that in comparison to the reference category of newborns devoid of both skilled and unskilled PNC, there have been lesser chances of neonatal mortality. This calls for accelerating the pace of new initiatives such as the India Newborn Action Plan (INAP) and strengthening existing initiatives such as *Janani Suraksha Yojana*, *Janani Shishu Suraksha Karyakram* and *Navajat Shishu Suraksha Karyakram* (NSSK) [31] along with building capacity of a skilled healthcare workforce in the high focus states. This is one of the very few studies that attempt to explore the relationship between timely postnatal care of neonates and early neonatal and neonatal mortality in India, especially by type of service provider among high and non-high focus states. Compared to the absence of PNC, services provided by unskilled and skilled providers were found to be protective for neonatal mortality, in that order. The lack of a skilled healthcare workforce had a profound effect on neonatal mortality among high focus states. The findings highlight the quality of care expected to be delivered by skilled healthcare providers and underline the scope for building their capacity to handle the influx of complicated maternal and newborn cases.

There are some limitations to the study. NFHS-4 as utilized by the study does not capture the nuances of obstetric complications and conditions in which a particular maternal and childcare service was pursued along with other structural and functional mechanisms relevant to the quality of care. This is important to consider when implications regarding a skilled and unskilled healthcare workforce have to be discussed. NFHS is also a cross-sectional survey, which limits the scope of establishing causality among variables. Also, the recollection of services (day of PNC provided and type of provider) received was for 5 years preceding the survey and there may be some inaccuracy in this also. As mentioned earlier, data collected limits in-depth information about neonatal deaths occurring within the first 48 hours and hence the implications drawn are devoid of the scope for the remedial measures that can be taken to ensure the survival of the newborn in the first 24 hours of birth.
